# The role of stromal immune microenvironment in the progression of ductal carcinoma in situ (DCIS) to invasive breast cancer

**DOI:** 10.1186/s13058-021-01494-9

**Published:** 2021-12-24

**Authors:** Anna Niwińska, Wojciech P. Olszewski

**Affiliations:** 1 Department of Breast Cancer and Reconstructive Surgery, Maria Sklodowska-Curie National Research Institute of Oncology, Roentgen 5 Str., 02-781 Warsaw, Poland; 2 Pathology Department, Maria Sklodowska-Curie National Research Institute of Oncology, Warsaw, Poland

**Keywords:** Breast cancer, DCIS, Immune microenvironment, Stromal cells, CD20, CD138, Syndecan-1, CD163, CD4, CD8

## Abstract

**Aim:**

The first aim of the study was to compare the scores and types of stromal immune cells in 30 patients with primary DCIS and in the same patients after invasive breast recurrence in order to assess possible differences in both during tumor progression. The second aim was to evaluate possible differences in stromal cells of 30 patients with primary DCIS before progression and in the control group of 11 DCIS patients without recurrence during long-term follow-up.

**Material and methods:**

Evaluation of tumor-infiltrating lymphocytes (TILs) and immunohistochemical stains for immune cell markers CD4, CD8, CD20, CD138, FOXP3, CD163 and TGF beta was performed on the stroma of primary DCIS before progression, invasive breast cancer of the same patients after progression and DCIS without progression.

**Results:**

The comparison of stromal cells in 30 patients with initial DCIS and its invasive recurrence revealed an increased level of CD20 + immune cells (median score 5% vs. 17%, respectively, *p* < 0.001) and CD163 + cells (median score 1% vs. 5%, respectively, *p* < 0.001) in invasive breast cancer. The comparison of stromal cells in 30 patients with initial DCIS before recurrence and the control group of 11 patients with DCIS without recurrence showed statistically significant difference for CD138 + cells, which were more prevalent in patients with worse prognosis (median score 0 vs. 2%, respectively, *p* < 0.001). No similar relationship was found for the other tested cells as well as for TGF-beta.

**Conclusions:**

CD138 + immune cells that were more prevalent in patients with a worse prognosis should be explored in further studies to confirm or exclude their role as a potential biological marker of DCIS invasive recurrence.

**Supplementary Information:**

The online version contains supplementary material available at 10.1186/s13058-021-01494-9.

## Background

DCIS (ductal carcinoma in situ) is a growing problem for oncologists due to an increasing number of patients with this disease and different ways of treatment of those patients. There is a clinical need to distinguish DCIS lesions with a high potential for invasive recurrence from those which are essentially benign in order to perform appropriate, more or less aggressive, tailored treatment. Known clinical and pathological parameters like age, tumor size, nuclear grade, *comedo* necrosis, resection margins still do not allow to accurately predict the risk of recurrence [1].

The analysis of genetic events in DCIS and invasive breast cancer revealed the lack of genomic and transcriptomic differences between the two [2]. There is growing evidence that changes in the stromal tissues surrounding DCIS play an integral role in breast tumor progression [1]. There are no data showing that the immune cells present in breast tumors can influence growth and metastasis [3]; however, the immune microenvironment in DCIS and its significance are not well established [1, 4, 5]. The mechanism by which DCIS progresses to invasive carcinoma is not well understood, but it is thought to be a complex process driven by tumor cells and tumor microenvironment [1, 4, 5]. A few studies have reported that dense tumor-infiltrating lymphocytes (TILs) in DCIS are associated with more aggressive clinical features and an increased risk of progression, while some TIL subsets in DCIS have been linked to tumor recurrence [6–10].

In invasive breast cancer, CD4 + T helper cells, CD8 + CTL cells, tissue-resident T cells, B cells, Natural Killer (NK) cells, M1 macrophages, and dendritic cells (DC) are protective against tumor growth (tumor-suppressing immune cells). They act through the production of cytokines that inhibit tumor development, secrete cytotoxic granules that trigger tumor apoptosis (CD8 + T cells) or secrete tumor-specific antibodies that eliminate tumor cells (B cells). Conversely, CD4 + FOX3 + TH2 cells (T reg = regulatory T cells), M2 macrophages, and myeloid-derived suppressor cells (MDSCs) suppress anti-tumor immune counterparts and drive tumor growth. These cells release immune-inhibitory cytokines, like TGF beta [11].

Unlike in invasive breast cancer, immune regulation in DCIS is not well established and search for a potential immunological prognostic marker of DCIS progression is now of special interest.

## Aim

The first aim of the study was to compare the composition of stromal immune cells and growth factor TGF-beta in primary DCIS and its ipsilateral invasive recurrence in order to evaluate possible changes in microenvironment during tumor progression.

The second aim was to detect possible changes in stromal immune cells in both groups of primary DCIS: with and without recurrence during long-term follow-up.

## Material

The study was retrospective in character. In 737 consecutive patients with DCIS treated in our institution between the years 1996–2011, with median time of observation 120 months (range 114–126 months), 68 recurrences were detected. Out of those 68 cases, 30 cases with “true recurrence” (localized in the tumor bed or in the same quadrant up to 5 cm from the tumor bed) [12, 13] as invasive breast cancer and biological subtype HER2-positive and luminal HER2-negative were selected.

**Fig. 1:**
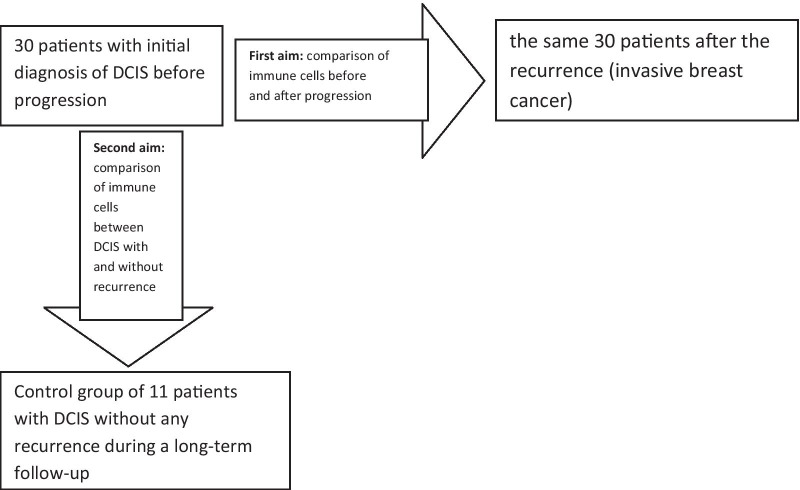
The scheme of the study

Formalin-fixed paraffin-embedded blocs (FFPE) from initial DCIS and invasive recurrence from 30 patients were analyzed to compare potential differences in the number of particular subdivisions of immune cells and the presence of TGF-beta. In the second part of the study, the control group of 11 DCIS without any recurrence during median time of observation 10 years (range 3–19) was selected in order to compare it with the group of 30 DCIS with further recurrence to reveal potential differences in stromal immune cells between both DCIS groups with a different outcome. The control group of 11 DCIS was matched to the group of 30 DCIS in terms of clinical and histological features, especially the biological subtype. The scheme of the study is presented in Fig. 1.

## Methods

Histological material for DCIS and invasive breast cancer came from postoperative material. FFPE neoplastic tissue intended for study came from routine diagnostic material. Cold ischemia times were below 1 h. Formalin fixation times were 48 to 72 h. Tumor areas with the most cellular (desmoplastic) stroma were selected. In cases of DCIS, the stromal area was defined as the special stroma surrounding the DCIS-affected ducts [14–16]. Sections for hematoxylin–eosin staining for histological verification and evaluation of tumor-infiltrating lymphocytes (TILs) in all cases were prepared, and sections for immunohistochemical staining (CD4, CD8, CD20, CD138, FOX P3, CD163, TGF beta) were obtained from each selected paraffin tissue block (in situ, the corresponding infiltrative recurrences, and the control group) to determine immune cell subsets. Immunohistochemical stains were prepared according to producer instructions: TGF beta 1,2,3 monoclonal antibody, Invitrogen; CD163, MRQ-26, Pathosolutions; Anti-Hu FOXP3, eBioscience, Invitrogen; CD4 (4B12), DAKO; CD8 (C8/144B), DAKO; CD20 (L26), DAKO; CD138 (MI15) DAKO. A total of 497 immunohistochemical stains were performed and evaluated in 71 sets (30 carcinomas in situ, 30 infiltrating carcinomas and 11 control carcinomas in situ). TILs were assessed in 71 cases.

Evaluation of slides: Evaluation of tumor-infiltrating lymphocytes (TILs) and areas of evaluation were defined. Stomal desmoplastic tissue within tumor area excluded of necrosis and fibrosis was area of evaluation. The denominator used to determine the percent of stromal TILs is the area of stromal tissue (i.e., area occupied by mononuclear inflammatory cells over total intratumoral stromal area), not the number of stromal cells (i.e., fraction of total stromal nuclei that represent mononuclear inflammatory cell nuclei).

Evaluation of immunohistochemical stains CD4, CD8, CD20, CD 138, CD163, TGF beta, FOX P3 was performed on immunological cells present in tumor stroma defined analogically to TILs evaluation. Population of all immunological cells is usually higher than TILs alone. Each evaluated immunohistologically antibody is denominated by percentage of total intratumoral stromal area defined as above. Immunological cells and their nuclei are usually smaller than tumor cells. They also often lie in tightly packed groups, which definitely make counting individual cells hard or even impossible to perform properly. To avoid these problems, we decided to count only area covered by IHC-stained cells in manners similar to TIL-counting instructions. We estimated the percentage of area of stromal tissues bordered by tissue bordered by DCIS structures, which was covered by stained cells (which were brown and contrasting with usually light stained stromal tissue—which is white, light blue, or pink in most cases). Sometimes, there were staining present on neoplastic cells (both in situ and invasive carcinoma. No matter intensity or proportion of stained tumor cells in such cases, tumor cells were not included to the study. Therefore, the final score is presented in percentage. Sometimes, positive IHC stain was visible in tumor cells. In such cases, only stromal cells’ stain was evaluated. In cases where IHC stain present in stromal tissue was seen in differently shaped cells (but not neoplastic cells), e.g., oval, round and spindle—area of all these types of cells was counted to the final score. Microphotographs of H&E and corresponding IHC CD138 stains as an example pictures that were analyzed in this study are presented in Additional file 1.

TILs and Immunohistochemical evaluation were performed in all cases using optical microscope (visual estimation). All cases were scored visually by pathologist.

This study was approved by the institutional review board. All procedures performed in studies involving human participants were in accordance with the ethical standards of the institutional and/or national research committee and with the 1964 Helsinki Declaration and its later amendments or comparable ethical standards. Because of the retrospective design of the analysis, requirement for obtaining an informed consent of participants included in the study was exempted.

### Statistical analysis

Standard descriptive statistics tools were used to describe the material. The nonparametric Wilcoxon test and the Mann–Whitney test were used for testing dependent and independent continuous variables, respectively. In the case of independent binary variables, the Chi-square test was applied, and in the case of dependent variables, the compatibility test was used. All tests were performed at the level of 0.1.

## Results

The baseline characteristics of the two DCIS groups—with a true recurrence and without any recurrence during long-term follow-up—are presented in Table 1. Patients from both groups were well balanced and did not differ significantly as regards clinical and histological features.

**Table 1:** Characteristics of initial DCIS of 30 patients in whom invasive local true recurrence was detected, the same 30 patients after invasive recurrence and 11 patients with DCIS without local recurrence during 15 years of observation

Feature	30 patients with initial DCIS before local recurrence, number (percentage)	The same 30 patients after invasive breast cancer recurrence, number (percentage)	11 patients without any recurrence, number (percentage)
Median age (range)	56 (44–75)	67 (48–83)	54 (49–70)
Median size of DCIS in mammography, (range) in mm	14 (5–70)	16 (2–30)	13 (9–40)
*Estrogen receptor*			
Positive	23 (77)	23 (77)	8 (73)
Negative	7 (23)	7 (23)	3 (27)
*HER2 receptor*			
Positive	8 (27)	7 (23)	5 (45)
Negative	22 (73)	23 (77)	6 (55)
*Biological type*			
HER2-positive ER/PgR-negative	7 (24)	5 (17)	3 (27)
HER2-positive ER/PgR-positive	1 (3)	2 (7)	2 (18)
Luminal A HER2-negative &	15 (50)	14 (47)	4 (37)
Luminal B HER2-negative#	7 (23)	8 (26)	2 (18)
Triple-negative	–	1 (3)	–
*Type of detection*			
Mammographically only	29 (97)	22(73)	10 (90)
Clinically	1 (3)	8 (27)	1 (10)
*Histological grade*			
G1	5 (17)	4 (13)	2 (18)
G2	14 (46)	16 (53)	4 (37)
G3	11 (37)	10 (33)	5 (45)
*Comedo necrosis*			
Yes	11 (37)	–	5 (45)
No	19 (63)		6 (55)
Median narrowest surgical margin (mm)	5 (1–30)	–	8 (1–10)
*Type of treatment*			
Breast-conserving surgery*	12 (40)	–	8 (72)
Breast-conserving treatment**	15 (50)	2 (7)	3 (27)
Mastectomy	3 (10)	28 (93)	0
Adjuvant hormonal therapy	0	–	0
Recurrence-free survival, median (range)	–	8 years (2–19)	–
*Localization of recurrence*			
In tumor bed	20 (67)	20 (67)	–
In the same quadrant up to 5 cm from tumor bed	10 (33)	10 (33)	–
*Presence of DCIS in invasive recurrence*			
Yes	–	4 (13)	–
No	–	26 (87)	–
*Lymph node metastases in invasive recurrence*			
Yes	–	2 (7)	–
No	–	28(93)	–

&- ER + PgR + HER2-Ki 67 <  = 20%; #- ER + PgR + HER2-Ki 67 > 20%

*Breast-conserving surgery = local excision without radiation therapy

**Breast-conserving treatment = breast-conserving surgery + radiation therapy

### Total stromal TILs’ infiltration (without division into CD4, CD8, etc.)

Table 2 presents the scores of total stromal TILs. The comparison of the level of TILs in 30 patients with primary DCIS before the recurrence (Table 2, column 2) and in the same patients after the detection of invasive breast cancer (Table 2, column 3) revealed a statistically significant increase in the score of TILs after the recurrence (10%, range 1–15 in DCIS vs. 15%, range 2–70 in invasive breast cancer, *p* = 0.003). An increase in the score of TILs was observed in almost all (29/30) patients.

**Table 2:** Percentage of stromal TILs in the group of 30 patients before and after recurrence (columns 2,3, respectively) and in 11 patients with DCIS without local recurrence (Column 4)

	Initial scores of stromal TILs in 30 patients with initial DCIS before recurrence	Scores of stromal TILs in 30 patients after detection of invasive recurrence	Scores of stromal TILs in 11 patients without recurrence
Median (range)	10% (1–45)	15% (2–70)	8% (1–35)
*Scores of TILs depending on biological subtypes*			
HER2-positive	5% (1–25)	5% (2–40)	8% (1–35)
Luminal HER2-negative	7% (1–45)	20% (5–70)	10% (2–20)
*Scores of TILs depending on estrogen receptor*			
ER-positive	7% (1–45)	20% (2–70)	8% (2–25)
ER-negative	10% (1–25)	5% (2–40)	5% (1–35)

The comparison of 30 patients with an initial DCIS diagnosis with further progression (Table 2, column 2) with 11 other DCIS patients without the recurrence at any time (Table 2, column 4) revealed that there were no statistically significant differences in the percentage of TILs in both groups in general (10%, range 1–45 vs. 8%, range 1–35, *p* = 0.988).

### Subsets of stromal cells

**Fig. 2:**
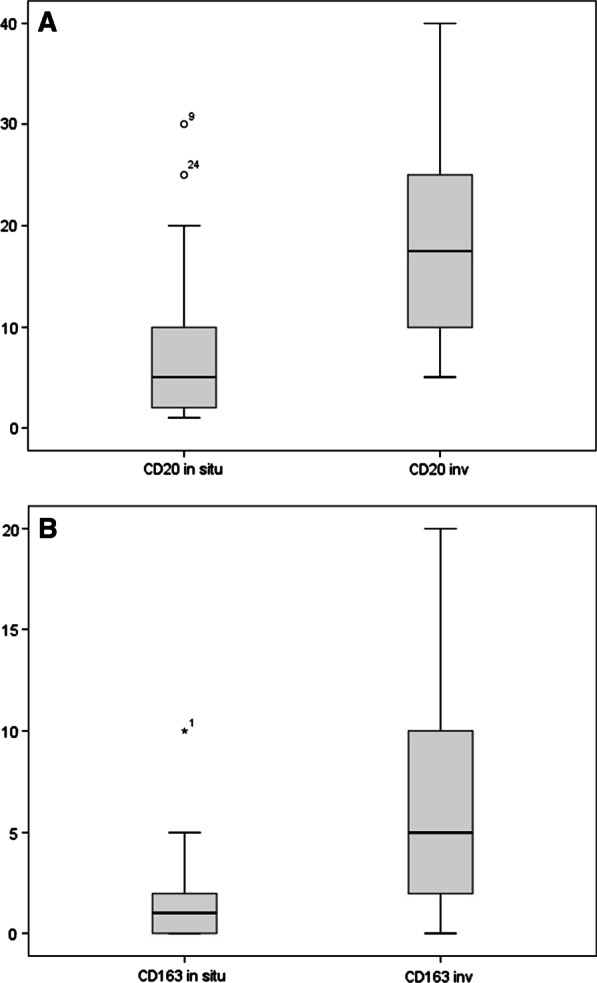
**A** The differences in the scores of CD20 + immune cells in the group of 30 patients with primary DCIS and the same 30 patients with ipsilateral breast recurrence (*p* < 0.001). **B** The differences in the scores of CD163 + immune cells in the group of 30 patients with primary DCIS, the same 30 patients with ipsilateral breast recurrence (*p* < 0.001)

**Fig. 3:**
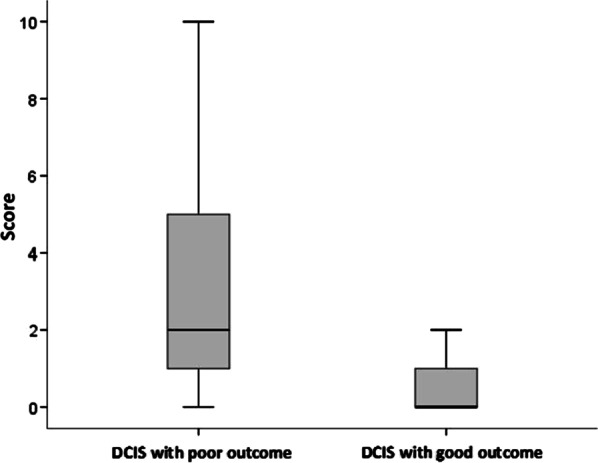
The differences in the scores of CD138 + immune cells in the group of 30 patients with primary DCIS and in 11 patients with primary DCIS without local recurrence (*p* < 0.001)

The differences in the level of particular immune cells and growth factor TGF-beta in the group of 30 patients with primary DCIS and the same 30 patients with ipsilateral breast recurrence are presented in Table 3 and Fig. 2, respectively. The differences in the scores of particular immune cells and growth factor TGF-beta in the group of 30 patients with primary DCIS and in 11 patients with primary DCIS without local recurrence are presented in Table 4 and Fig. 3, respectively. The distribution of the scores of CD138 in the group of 30 patients with primary DCIS and in 11 patients with primary DCIS without local recurrence is presented in Additional file 2: Table S4A. The percentage of patients with a positive reaction to immune tests (more than 0, at least 1%) in the group of 30 patients with primary DCIS, the same 30 patients with invasive breast recurrence (columns 2, 3) and in 11 patients with primary DCIS without local recurrence (column 4) are presented in Table 5.

**Table 3:** The differences in the scores of particular immune cells and growth factor TGF-beta in the group of 30 patients with primary DCIS and in the same 30 patients with ipsilateral breast recurrence

Type of immune cell	Initial scores of immune cells in 30 patients with initial DCIS before recurrence median (range)	Scores of immune cells in 30 patients after detection of invasive recurrence median (range)	*p*-value
**CD20+**	**5% (1**–**30)**	**17.5% (5**–**60)**	** < 0.001**
**CD 163+**	**1% (0**–**10)**	**5% (0**–**20)**	** < 0.001**
CD 138+	2% (0–30)	5% (0–20)	0.100
CD4+	1% (0–4)	1% (0–4)	0.483
CD8+	2% (1–10)	2% (0–5)	0.593
FOXP3+	0% (0–3)	1% (0–3)	0.439
TGF-Beta (percentage of patients with the positive reaction)	30%	80%	0.426

Bold indicates statistical significance level alpha = 0.1

**Table 4:** The differences in the scores of particular immune cells and growth factor TGF-beta in the group of 30 patients with primary DCIS and in 11 patients with primary DCIS without local recurrence

Type of immune cell	Initial scores of immune cells in 30 patients with initial DCIS before recurrence median (range)	Scores of immune cells in 11 patients without recurrence median (range)	*p*-value
CD20+	5% (1–30)	5% (1–15)	0.942
CD 163+	1% (0–10)	1% (0–3)	0.942
**CD 138+**	**2% (0–30)**	< 1% (0–2)*	** < 0.001**
CD4+	1% (0–4)	0% (0–2)	0.287
CD8+	2% (1–10)	1% (1–10)	0.717
FOXP3+	0% (0–3)	0% (0–1)	0.513
TGF-Beta (percentage of patients with positive reaction)	30%	18%	0.371

Bold indicates statistical significance level alpha = 0.1

*55% cases no stain, 45% cases 1–2% of stromal area

**Table 5:** The percentage of patients with a positive reaction (more than 0, at least 1%) to immune tests in the group of 30 patients with primary DCIS, in the same 30 patients with ipsilateral breast recurrence (columns 2, 3, respectively) and in 11 patients with primary DCIS without local recurrence (column 4)

Type of immune cells	Thirty patients with the initial DCIS before recurrence (%)	Thirty patients after detection of invasive recurrence (%)	Eleven patients without recurrence (%)
CD20+	30/30 (100)	30/30 (100)	11/11 (100)
CD163+	22/30 (73)	26/30 (87)	8/11 (73)
CD138+	26/30 (87)	30/30 (100)	5/11 (45)
CD4+	19/30 (63)	23/30 (77)	5/11 (45)
CD8+	30/30 (100)	27/30 (90)	11/11 (100)
FOXP3+	11/30 (37)	20/30 (67)	5/11 (45)
TGF-beta	9/30 (30)	24/30 (80)	2/11 (18)

#### CD20+

Statistically significant correlation was found between the level of CD20 + cells in the same 30 patients before and after invasive recurrence of DCIS (median score of 5% vs. 17% stromal TILs, respectively, *p* < 0.001). Growing scores of CD20 + cells were observed after the invasion of breast cancer.

There were no differences in the CD20 + level between 30 DCIS patients with further recurrence and 11 patients without failure (median score of 5% vs. 5% of stromal TILs, respectively, *p* = 0.942).

#### CD163+

A statistically significant correlation was found between the level of CD 163 + cells in 30 patients with the primary DCIS and its invasive breast cancer recurrence. Median score of CD163 + cells in 30 patients with primary DCIS was 1%, and after the invasive recurrence, it was 5% (*p* < 0.001). An increase in CD163 + immune cells was observed in 28 of 30 patients.

There were not statistically significant differences in the level of CD163 + cells between 30 primary DCIS patients with further recurrence and 11 primary DCIS patients without failure (median score 1% vs. 1% of stromal immune cells, respectively, *p* = 0.942).

#### CD138+

There were no significant differences between the rate of CD138 + cells in DCIS and its recurrence (median score 2% vs. 5%, respectively, *p* = 0.1).

However, a statistically significant difference in the level of CD 138+ between the two groups of DCIS patients, with and without the recurrence, was observed. In the primary DCIS patients without any recurrence, the scores of CD 138 + cells were very low and in the group of primary DCIS patients with further recurrence the percentage, and the range of these immune cells were statistically significantly higher (0%, range 0–2 vs. 2%, range 0–70, respectively, *p* < 0.001).

#### CD4+

There were no statistically significant differences in the score of CD4 + T lymphocytes between 30 DCIS with recurrence and the subsequent invasive breast cancer (median 1% vs.1%, respectively, *p* = 0.483).

There were no statistically significant differences in the score of CD 4 + immune cells between 30 DCIS patients with further recurrence and 11 patients without failure (median 1% vs. 0%, respectively, *p* = 0.287).

#### CD8+

There were no statistically significant differences in the score of CD8 + T lymphocytes between 30 DCIS patients and its invasive breast cancer (median 2% vs.2%, respectively, *p* = 0.593).

There were no statistically significant differences in the score of CD 8 + T lymphocytes between 30 DCIS patients with further recurrence and 11 patients with DCIS without any failure (median 2% vs. 1%, respectively, *p* = 0.717).

#### FOXP3+

There were no statistically significant differences in the score of FOXP3 + regulatory T lymphocytes between 30 DCIS patients with further recurrence and its invasive breast cancer (median 0% vs.1%, respectively, *p* = 0.439), but a modest increase in the score of those cells after invasion was observed.

There were no statistically significant differences in the score of FOXP3 + cells between 30 DCIS patients with a poor outcome and 11 patients with DCIS with a good outcome (median 0% vs. 0%, respectively, *p* = 0.513).

#### Tumor growth factor beta (TGF-beta)

The percentage of patients with a positive reaction to TGF-beta was assessed. The growing number of patients with a positive reaction to TGF-beta after recurrence was detected. In 30 patients with primary DCIS, it was 30% and in patients with its invasive recurrence this was 80%; however, the difference was not statistically significant (*p* = 0.426).

There was no statistically significant difference in the percentage of patients with a positive reaction between 30 primary DCIS patients with further recurrence and 11 DCIS patients without any failure (median 30% vs. 18%, respectively, *p* = 0.371).

### Correlation between immune cell subsets and their relationship with clinicopathologic features of pure DCIS

Biological subtypes were assessed by the expression of estrogen receptor (ER), progesterone receptor (PgR), HER2 receptor and Ki- 67 protein in immunohistochemistry (IHC). Two groups of cases were distinguished: HER2-positive (ER+/PGR + HER2+ and ER-/PgR- HER2 +) and HER2-negative (ER+/PgR + HER2-). A statistical analysis did not reveal any relationship between the score of CD20+, CD163+ and CD138 + immune cells and pathological features of DCIS: histological grade (*p* = 0.98, *p* = 0.621, *p* = 0.192, respectively) and biological subtype HER2-positive vs. HER2-negative (*p* = 0.848, *p* = 0.099, *p* = 0.217, respectively).

## Discussion

### Selection criteria of DCIS cases

The most important criterion in the selection of patients with DCIS recurrence for this study was a recurrence in the form of an invasive cancer with no or the least DCIS component. The second criterion was “true recurrence,” which was to ensure that the assessed changes in the tumor stroma actually result from the transformation of the very same tumor. In addition, to distinguish a (de novo) second DCIS with invasive breast cancer from a progression of the first primary DCIS, the convergence of the biological subtypes of initial DCIS and invasive recurrence was compared.

### First aim of the present study: comparison of immune cell subsets between primary DCIS and its invasive recurrence

The first aim of the present study was to detect potential differences in the immune stromal microenvironment in 30 patients with primary DCIS and its invasive breast recurrence. A statistically significant correlation was found between the scores of CD20+ (*p* < 0.001) and CD163 + cells (*p* < 0.001). No similar relationship was found for the other tested cells as well as for TGF-beta. Our results were consistent with the findings of other studies [3, 4, 10, 11, 17].

#### CD20+, CD 163+ immune cells

CD20 is mainly expressed on B cells and in a subpopulation of T lymphocytes and follicular dendritic cells. B lymphocytes are CD20 + adaptive immune cells, which trigger humoral immunity through the production and secretion of antibodies which recognize specific tumor’s antigens and inhibit the functionality of the receptor. Additionally, antibodies are able to signal other cancer killing cells to eliminate tumor cell population [11]. In retrospective studies, B cells have been associated with favorable prognosis in invasive breast cancer [18]. Though this supports an anti-tumor role for B cells and antibodies, other studies have associated them with poor prognostic factors in breast cancer because human breast cancer cells can induce a regulatory phenotype in B cells, initiating the production of the transforming growth factor beta (TGF-beta), a cytokine that stimulates CD4 + T cells to become immunosuppressive T regulatory cells [19].

CD163 is expressed by tissue macrophages and monocytes. Tumor-associated macrophages (TAM) and their infiltration accompany a worse prognosis in ER- and ER + breast tumors [20]. TAM exerts pro-tumor effects: stimulate angiogenesis, metastasis, suppression of adaptive immunity and production of matrix proteins. The highest risk of DCIS recurrence is observed in patients with elevated M2 macrophages, suggesting that M2 macrophages may abrogate the tumor-fighting T cell function [7, 11].

#### CD4 +, CD8 +, CD 138 + immune cells

In the present study, we did not find any relationship between CD4+, CD8+ and CD 138+ in primary DCIS and invasive breast cancer recurrence in the same patients. Similarly, we did not observe statistically significant differences between the scores of FOXP3 + immune cells in both groups; however, increase in the number of those cells after invasive progression was revealed. It was very difficult to analyze the role of FOXP3 + regulatory lymphocytes in the progression of DCIS because the score of these cells in the stroma in both groups was very low.

CD8 is found on a T cell subset of normal cytotoxic/suppressor cells and natural killer cells. CD8 + cytotoxic T lymphocytes are typically associated with anti-tumor immunity, although their activity can be suppressed in the tumor microenvironment [21]. They are important immune prognostic markers for the outcome of TNBC and HER2-positive invasive breast cancer and correlate positively with improved survival [22].

CD4 is expressed on the surface of T helper cells, monocytes, macrophages, and dendritic cells. There are a number of subsets of CD4 + T cells that have different functions within the tumor. T helper 1 (TH1) cells represent a subset of the CD4 + T cell population that typically expresses high levels of interferon gamma, which acts to limit tumor growth, promote antigen presentation and active macrophages M1. Contrary to TH1, the helper cells type 2 (CD4 + TH2) secrete cytokines which inhibit T-cell mediated cytotoxicity, stimulate macrophages and promote a pro-tumorigenic environment [4, 11, 21].

FOXP3 is specifically expressed in T regulatory cells (Tregs), including CD4^+^ CD25^high^Treg cells and CD8^+^ CD25^high^ Treg cells. T regulatory lymphocytes are both CD4 + and FOXP3 + TH2 and have immunosuppressive functions. Normally, they help to protect against autoimmunity. In breast cancer, they are associated with poor prognosis as they contribute to the pro-tumor immune response and assist the tumor in subsequent immune escape. T regs CD4 + FOXP3 + allow the progression of the tumor by expressing inhibitory factors that inhibit the anti-tumor TH1 response [21, 23]. Thus, CD4 + Th1 cells, CD4 + CTLs exert anti-tumor activity, whereas regulatory T cells, CD4 + TH2 cells show tumor-promoting activity [4, 21, 23].

Our results concerning CD4+, CD8 + and FOXP3 + cells differ from the findings reported in other studies. Kim et al. [4] showed, basing on 590 cases, that the immune microenvironment of DCIS is different from that of invasive cancer. CD4+, CD8 + and FOXP3 + immune cell infiltration was significantly higher in invasive breast cancer compared to pure DCIS (*p* < 0.001). The comparison of the dominance of CD4 + versus CD8 + cells in pure DCIS and invasive cancer revealed that the infiltration of CD4 + TILs was higher than of CD8 + in pure DCIS (*p* < 0.001), while the reverse was true in invasive breast cancer with the CD8 + TILs being the dominant subset (*p* = 0.006) [4]. This is consistent with other studies [24, 25], but we did not observe such a relationship in our study. The difficulty in drawing a conclusion as to the role of CD4 + in our analysis is that CD4 + TILs display a large degree of plasticity and the ability to differentiate into multiple subsets in response to microenvironmental cues. Actually, we did not know whether the detected CD4 + cells in the present study acted in favor of (TH1 subset) or against (TH2 subset) tumor growth [4]. Thus, in further studies, analyses of CD4+ subsets would be crucial in establishing their role in tumor progression.

In our study, a comparison was made of 30 DCIS and 30 recurred invasive breast cancers in the same patients. To our knowledge, the only article concerning the comparison of the same patients with primary DCIS and its ipsilateral breast recurrence was written by Kim et al. [4]. However, unlike in their study, in our study recurrence was reported in only 6 patients. In 4 cases, it was pure DCIS while in 2 invasive breast cancers. In those 6 patients, the high infiltration of FOXP3 + TILs was found to be associated with decreased recurrence-free survival (*p* = 0.002). However, CD4+ and CD8 + TIL infiltration did not show prognostic significance (*p* = 0.287). The relationship between the ratios of TILs FOXP3+ /CD8+, FOXP3+/CD4+ and CD4+/CD8+ and tumor recurrence was analyzed and only high FOXP3+/CD8+ and high FOXP3/CD4 + ratios were found to be associated with a decreased recurrence-free survival (*p* = 0.02 and *p* = 0.036, respectively) [4]. The findings of Kim et al. [4] concerning the analysis of a small group of primary DCIS and its ipsilateral breast recurrence were in agreement with our observations as regards CD4+, CD8+, and it seems that only such a type of comparison should be performed to study the relationship between immune cells subsets during disease progression.

### Second aim of the study: the search of immune cells that play a role in the transition of DCIS to invasive breast cancer

The second aim of the present study was to try to select the immune cells that play a role in the transition of DCIS to invasive breast cancer, but only those which would be detectable in primary DCIS before progression and could be useful clinical factors of a poor prognosis. Based on such biological prognostic factors, detected in initial pathology of excised DCIS, oncologists might be able to tailor DCIS treatment depending on the risk of relapse (adjuvant radiation therapy, adjuvant hormonal therapy, mastectomy). To search for such markers, the group of 30 DCIS with further recurrence (poor outcome) and 11 control DCIS without the recurrence (a good outcome) were compared. The two groups did not differ significantly as regards clinical and pathological risk factors. We did not observe significant differences in the scores of immune cells between DCIS with a bad and good outcome except for CD138 + cells, which were present in greater numbers in patients with DCIS with a poor outcome.

#### Stromal syndecan-1 expression CD 138+

Syndecan-1 (CD 138) is a transmembrane heparin sulfate proteoglycan, which acts as an extracellular matrix receptor, and is expressed on the surface of mature epithelial cells, precursor B cells and plasma cells [10, 26]. It is involved in many cellular functions, including cell–cell and cell–matrix adhesion. CD-138-clustered antibodies recognize syndecan-1. In some studies of breast cancer, almost all cases of metastatic breast cancer exhibited membranous immune reactivity for CD 138, and all cases of metastatic breast cancer also exhibited stromal reactivity [26]. The majority of epithelial neoplasms, both primary and metastatic, showed reactivity for CD138 for neoplastic cells and frequently also for stromal cells [26]. In other studies, weak expression in malignant ductal cells associated with extensive stromal staining has been described [27]. The functional significance of syndecan-1 expression in the setting of the tumor stroma of breast cancer remains to be elucidated. It has, however, been postulated to have implications for tumor cell growth [26]. As CD 138 interacts with heparin binding growth factors such as fibroblast growth factor-2, its accumulation in tumor stroma might contribute to angiogenesis, cell proliferation and migration, tumor pathogenesis and cell–matrix interactions [26]. Leivonen et al. [28] assessed the prognostic value of the immunohistochemical expression of syndecan-1 in 200 patients with invasive breast cancer. The study revealed that syndecan-1 was expressed in the epithelium in 61% and in the stroma in 67% of tumors. Epithelial expression of syndecan-1 was associated with a negative ER status and stromal syndecan-1 expression with a positive ER status. Stromal expression was found in 71% of ER-positive tumors and 53% ER-negative tumors (*p* = 0.02). The 10-year breast cancer-specific survival of patients with tumors not expressing stromal syndecan-1 was 83% compared with 66% for those with positive staining. Negative stromal staining was consistently associated with a more favorable survival. Patients with both epithelial and stromal expression had a 10-year survival of 56% as compared to 78% in patients with other expression pattern combinations (*p* < 0.002) [28]. However, in Cox multivariate analysis, only tumor size and axillary involvement were found to be significant predictors of breast cancer-specific survival. The authors conclude that concomitant expression of epithelial and stromal syndecan-1 seems to identify a group of patients with an unfavorable outcome in invasive breast cancer [28].

Little is known about the importance of immune stromal syndecan-1 in DCIS as a potential prognostic factor of invasive recurrence. In the study by Miligy et al. [10], more CD138 + plasma cells were observed in pure DCIS cases than in DCIS cases with invasion. In our study, the comparison of two subsets of DCIS, with and without recurrence, revealed stromal syndecan-1 CD 138 + plasma cells as the only immune parameter which may help predict recurrence.

To our knowledge, this is the first study in which a potential role of the stromal syndecan-1 as a potential biological marker of poor prognosis of DCIS is attempted to be defined. To confirm or reject our findings, further larger-series studies investigating the composition of immune cell subsets in DCIS are required to understand the role of individual TIL subsets in tumor progression.

### Strengths and limitations of the study

Firstly, in this project, a unique selection was made of 30 patients with pure DCIS and its subsequent invasive recurrence (was performed). This was possible due to the large database of 737 consecutive DCIS patients treated in our institution between the years 1996–2011 with a long-term follow-up. Secondly, only cases with invasive recurrence, localized in the tumor bed or in the same quadrant of the breast up to 5 cm from the tumor bed, were included in the analysis to be sure that the study concerns true recurrence and not second ipsilateral breast cancer. The third strength of the study was a careful choice of a control group with DCIS without recurrence. Based on the fact that clinical and histological factors are important in determining recurrence, the choice of similar cases in both groups seemed the best guarantee of reliable results. Fourthly, the time of observation of our patients was long and the choice of the control group after median 10 years of observation without local recurrence seems to be important. Finally, despite the fact that the scoring system for TILs subsets by immunohistochemistry on hematoxylin- and eosin-stained sections in DCIS has not been optimized yet, our pathologist (W.P.O.) performed and counted the scores of immune subsets based on the proposal of the International Immuno-Oncology Biomarker Working Group in which only stromal TILs were evaluated [12–14]. That is why the medians of particular TIL subsets in our study differed from those published in other papers [4].

This study has its limitations. First of all, it was a retrospective study. Moreover, the small size of the analyzed groups of patients made it impossible to perform a more reliable statistical analysis and that is why all the presented results, however interesting, should be interpreted with caution.

## Conclusions

The comparison of the scores of stromal immune cells in 30 patients with primary DCIS and its invasive recurrence revealed an increased level of stromal CD20 + lymphocytes B and CD163 + macrophages M2 in invasive breast cancer what is consistent with results reported by other authors. The comparison of the level of stromal cells in 30 primary DCIS before recurrence and the control group of 11 DCIS without recurrence during the long-term follow-up showed a statistically significant difference for CD138 + cells, which were more prevalent in patients with invasive breast cancer relapse. CD138 + cells should be analyzed in further DCIS studies as a potential prognostic biological marker of DCIS recurrence.

## Supplementary Information

**Additional file 1**. Microphotographs of H&E and corresponding IHC CD138 stains as an example pictures that were analyzed in the present study. **Figure S1A**. DCIS H&E stain, stromal tissue bordered by DCIS. **Figure S2A**. DCIS IHC CD138 strong stain, visible in stromal tissue bordered by DCIS. **Figure S3A**. Invasive breast carcinoma G1 H&E stain. **Figure S4A**. Invasive breast carcinoma G1 IHC CD138 strong stain. **Figure S5A**. Invasive breast carcinoma G2 H&E stain. **Figure S6A**. Invasive breast carcinoma G2 IHC CD138 faint stain. **Table S4A**. The distribution of the scores of CD138 in the group of 30 patients with primary DCIS and in 11 patients with primary DCIS without local recurrence.

## Data Availability

The datasets used and/or analyzed during the current study are available from the corresponding author on reasonable request.

## Abbreviations

DCIS: Ductal carcinoma in situ
ER: Estrogen receptor
HER2: Human epidermal growth factor receptor 2
TGF-beta: Tumor growth factor beta
TIL: Tumor-infiltrating lymphocyte
PR: Progesterone receptor
