# Supplementary material for: The role of stromal immune microenvironment in the progression of ductal carcinoma in situ (DCIS) to invasive breast cancer
Source: Breast Cancer Res. 2021 Dec 24;23:118. doi: 10.1186/s13058-021-01494-9 (PMC8710011; doi:10.1186/s13058-021-01494-9)
Supplement: Supplementary file 1 — Additional file 1. Microphotographs of H&E and corresponding IHC CD138 stains as an example pictures that were analyzed in the present study. Figure S1A. DCIS H&E stain, stromal tissue bordered by DCIS. Figure S2A. DCIS IHC CD138 strong stain, visible in stromal tissue bordered by DCIS. Figure S3A. Invasive breast carcinoma G1 H&E stain. Figure S4A. Invasive breast carcinoma G1 IHC CD138 strong stain. Figure S5A. Invasive breast carcinoma G2 H&E stain. Figure S6A. Invasive breast carcinoma G2 IHC CD138 faint stain. Table S4A. The distribution of the scores of CD138 in the group of 30 patients with primary DCIS and in 11 patients with primary DCIS without local recurrence. [file 13058_2021_1494_MOESM1_ESM.docx]

**Supplementary material**

Additional File nr 1. Microphofographs of H&E and corresponding IHC CD138 stains as an example pictures that were analyzed in the present study. File format DOC (Microsoft Word).

Figure 1A. DCIS H&E stain, stromal tissue bordered by DCIS.

Figure 2A. DCIS IHC CD138 strong stain, visible in stromal tissue bordered by DCIS.

Figure 3A. Invasive breast carcinoma G1 H&E stain.

Figure 4A. Invasive breast carcinoma G1 IHC CD138 strong stain.

Figure 5A. Invasive breast carcinoma G2 H&E stain.

Figure 6A. Invasive breast carcinoma G2 IHC CD138 faint stain.

Additional File nr 2.

Table 4 A. The distribution of the scores of CD138 in the group of 30 patients with primary DCIS and in 11 patients with primary DCIS without local recurrence. File format DOC (Microsoft Word).

Figure 1A. DCIS H&E stain, stromal tissue bordered by DCIS.


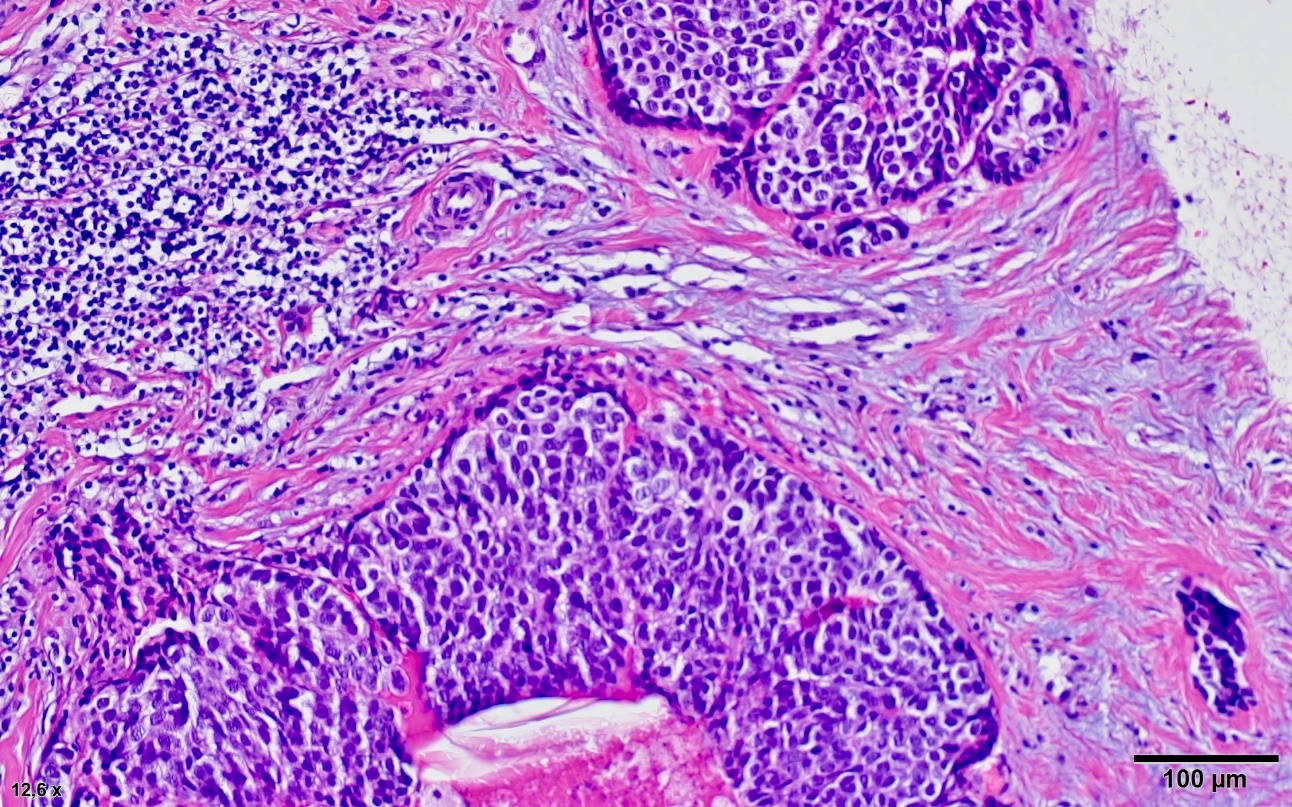


Figure 2A. DCIS IHC CD138 strong stain, visible in stromal tissue bordered by DCIS.


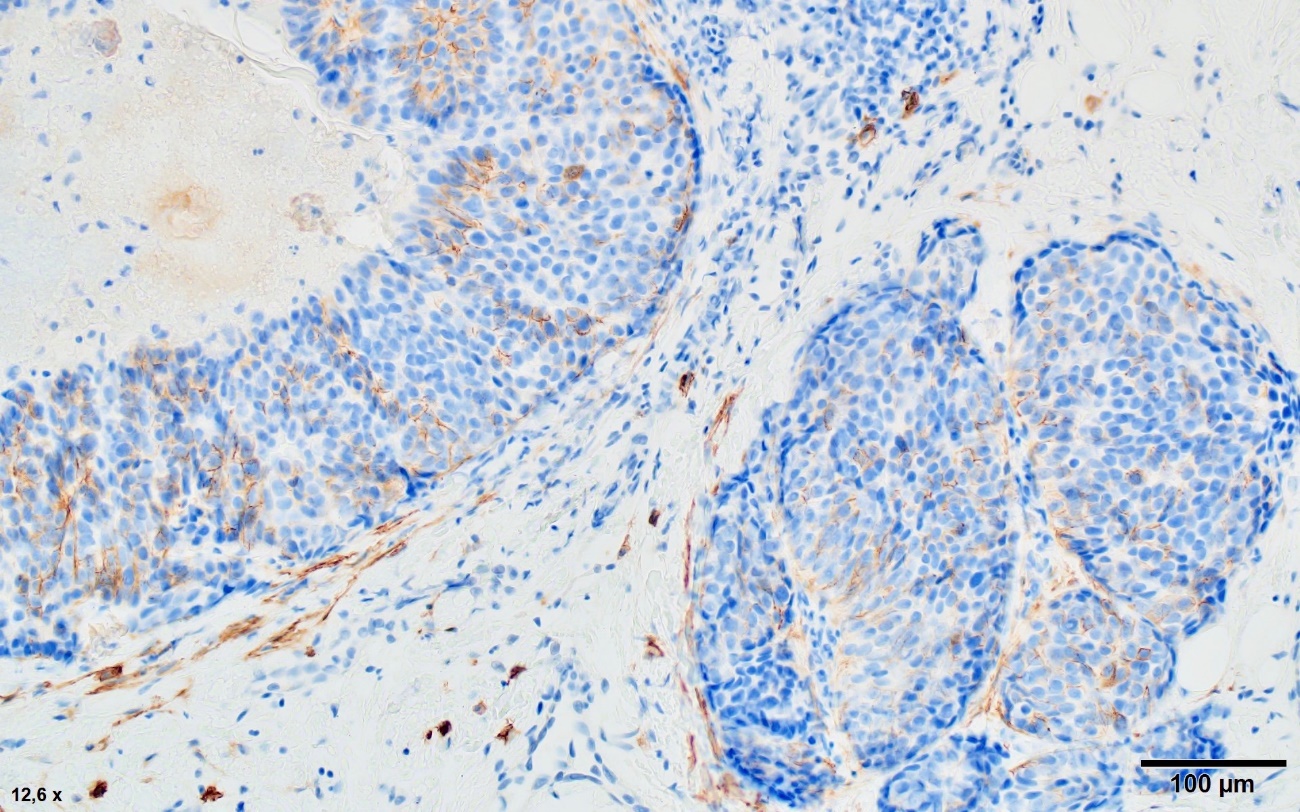


Figure 3A. Invasive breast carcinoma G1 H&E stain.


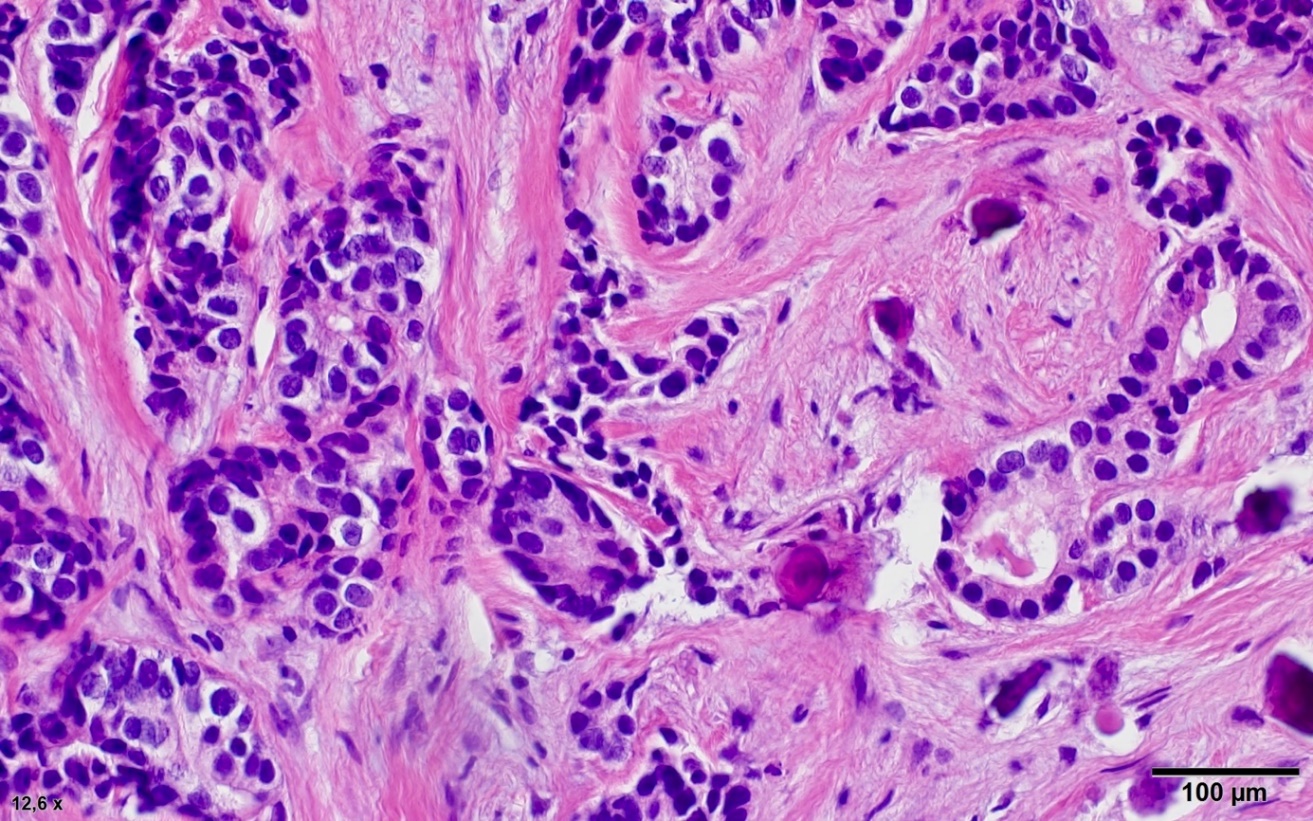


Figure 4A. Invasive breast carcinoma G1 IHC CD138 strong stain.


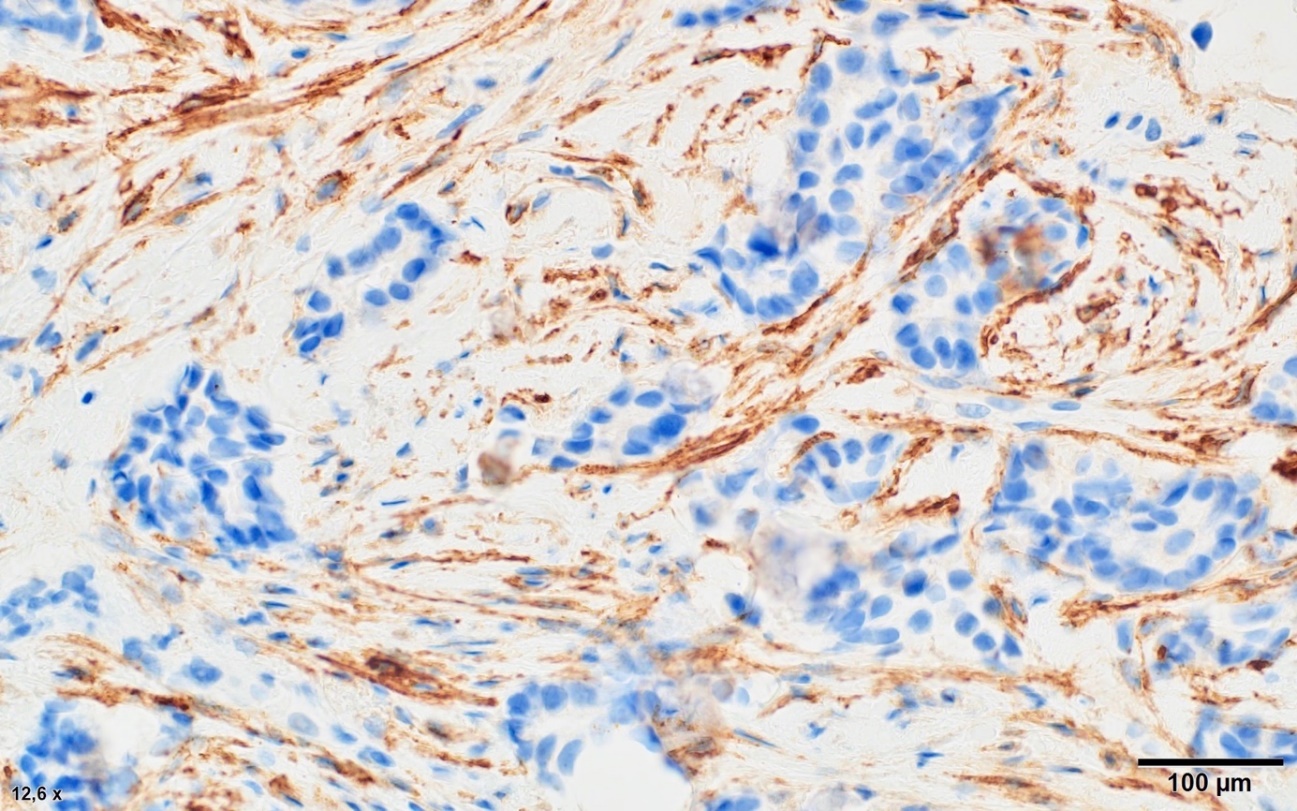


Figure 5A. Invasive breast carcinoma G2 H&E stain.


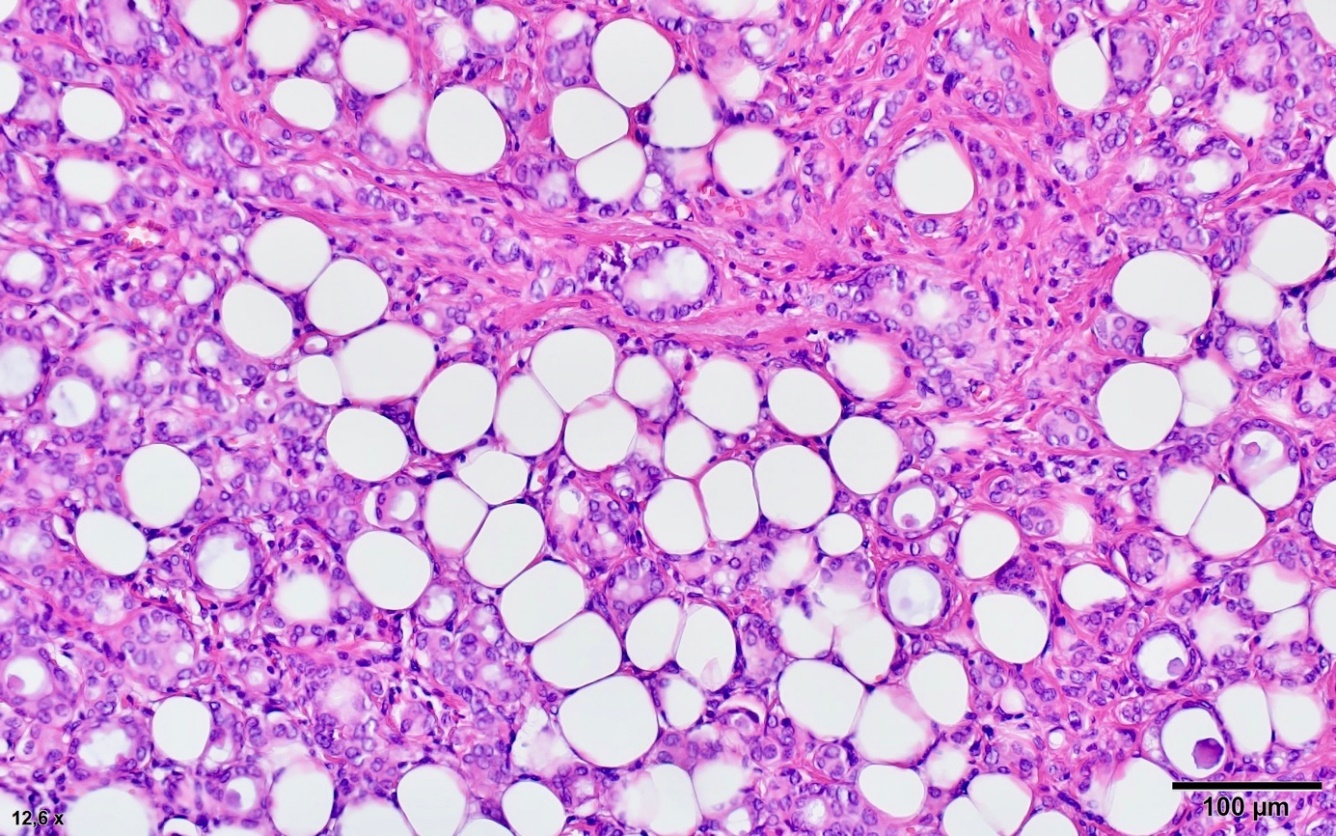


Figure 6A. Invasive breast carcinoma G2 IHC CD138 faint stain.


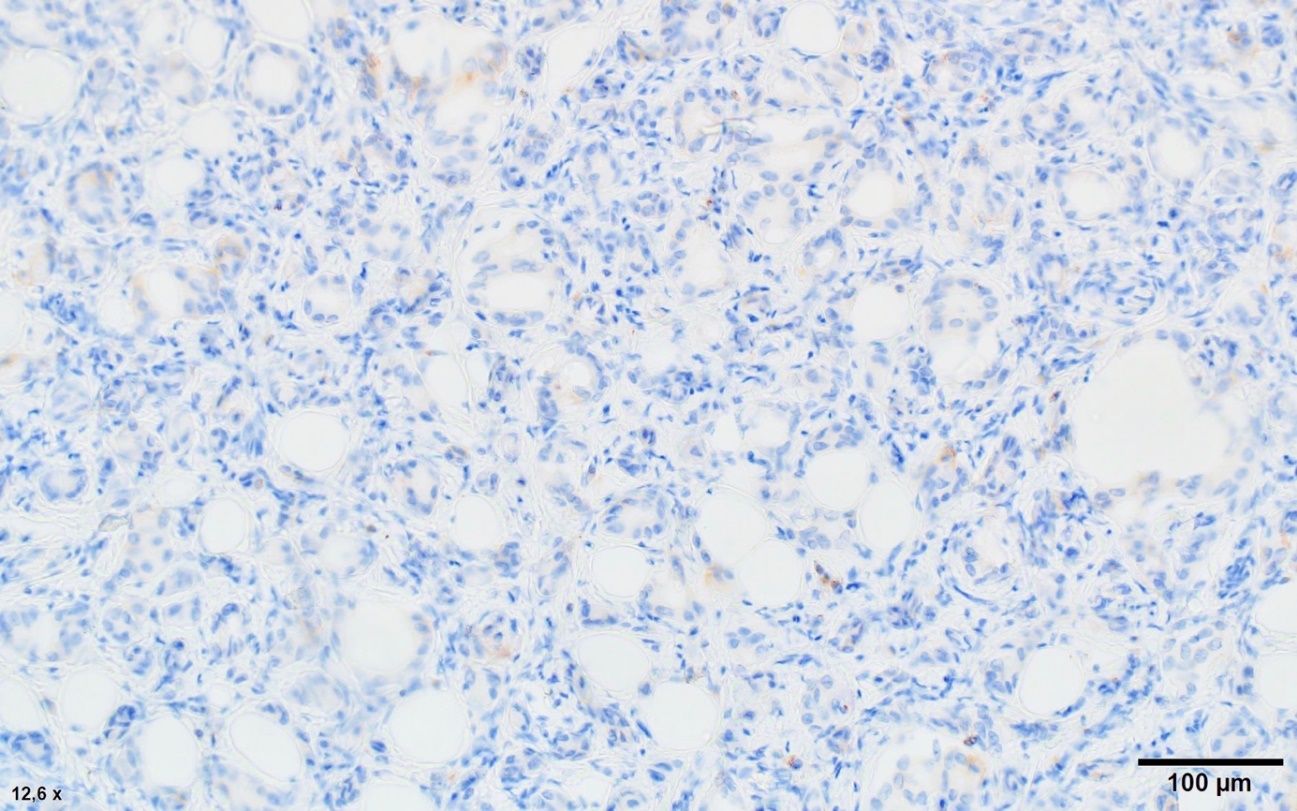


Additional File nr 2.

Table 4 A. The distribution of the scores of CD138 in the group of 30 patients with primary DCIS and in 11 patients with primary DCIS without local recurrence.

| Percentage of stromal area | CD138 immune cells 30 patients with initial DCIS **before recurrence**  Number of patients (%) | CD138 immune cells 11 patients **without recurrence**  Number of patients (%) |
| --- | --- | --- |
| **0%** | **4 (13.5)** | **6 (55)** |
| 1-2% | 13 (43.5) | 5 (45) |
| 3-5% | 8 (27) | 0 |
| 6-10% | 1 (3) | 0 |
| 11-20% | 2 (7) | 0 |
| 21-40% | 1 (3) | 0 |
| 41-70% | 1 (3) | 0 |
